# Alleviative effect of *Ruellia tuberosa* L. on NAFLD and hepatic lipid accumulation via modulating hepatic de novo lipogenesis in high‐fat diet plus streptozotocin‐induced diabetic rats

**DOI:** 10.1002/fsn3.1868

**Published:** 2020-09-09

**Authors:** Da‐Wei Huang, Yangming Martin Lo, Wen‐Chang Chang, Chia‐Yu Lin, Jou‐An Chen, James Swi‐Bea Wu, Wen‐Chung Huang, Szu‐Chuan Shen

**Affiliations:** ^1^ Department of Biotechnology and Food Technology Southern Taiwan University of Science and Technology Tainan City Taiwan; ^2^ Institute for Advanced Study Shenzhen University Shenzhen China; ^3^ Department of Food Science National Chiayi University Chiayi City Taiwan; ^4^ Graduate Program of Nutrition Science National Taiwan Normal University Taipei Taiwan; ^5^ Graduate Institute of Food Science and Technology National Taiwan University Taipei Taiwan; ^6^ Graduate Institute of Health Industry Technology Chang Gung University of Science and Technology Taoyuan Taiwan

**Keywords:** de novo lipogenesis, hepatic lipid accumulation, *Ruelliatuberosa* L., type 2 diabetes mellitus

## Abstract

*Ruellia tuberos*a L. (RTL) exhibits phytochemical activities and has been used as a folk medicine for curing diabetes mellitus in East Asia for decades. This study investigated the effect of RTL aqueous and ethanolic extracts on nonalcoholic fatty liver disease (NAFLD) and hepatic lipid accumulation in high‐fat diet (HFD) and streptozotocin (STZ)‐induced type 2 diabetes mellitus (T2DM) rats. Administration of RTL aqueous extract (RTLW) or ethanolic extract (RTLE) at dosage of 100 or 400 mg/kg body weight for 4 weeks was carried out in HFD/STZ‐induced T2DM rats. Liver weight, adipose (epididymal and perirenal adipose tissues) weight, hepatic triglyceride level, and de novo lipogenesis (DNL)‐associated protein expression were monitored after scarification. The results revealed that RTLW and RTLE reduced relative liver weight and relative fat weights in HFD/STZ‐induced T2DM rats. RTLW and RTLE also ameliorated NAFLD and hepatic triglyceride (TG) accumulation in diabetic rats. Moreover, hepatic DNL‐regulated enzymes such as sterol regulatory element‐binding protein‐1 (SREBP1) and fatty acid synthase (FAS) expression were significantly suppressed by RTLE (100 and 400 mg/kg body weight) in diabetic rats. The evidences of this study suggest that RTL possesses potential on alleviating NAFLD and lipid accumulation via regulating DNL in the liver of HFD/STZ‐induced T2DM rats.

## Graphical abstract

This study investigated the effect of RTL aqueous and ethanolic extracts on nonalcoholic fatty liver disease (NAFLD) and hepatic lipid accumulation in high‐fat diet (HFD) and streptozotocin (STZ)‐induced type 2 diabetes mellitus (T2DM) rats. Liver weight, adipose (epididymal and perirenal adipose tissues) weight, hepatic triglyceride level, and de novo lipogenesis (DNL)‐associated protein expression were monitored after scarification. The evidences of this study suggest that RTL possesses potential on alleviating NAFLD and lipid accumulation via regulating DNL in the liver of HFD/STZ‐induced T2DM rats.

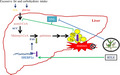

## 1 INTRODUCTION

Nonalcoholic fatty liver disease (NAFLD) has been associated with obesity, insulin resistance, type 2 diabetes mellitus (T2DM), and cardiovascular disease (Browning et al., 2004). Defined as the presence of macrovesicular steatosis by more than 5% in liver of individuals without alcohol intake (Loomba & Sanya, 2013), NAFLD develops when the rate of fatty acid uptake and de novo lipogenesis (DNL) exceeds the rate of fatty acid output such as fatty acid oxidation and secretion of very‐low‐density lipoprotein (VLDL) (Fabbrini, Sullivan, & Klein, 2010). De novo lipogenesis is the main metabolic pathway for converting excess carbohydrate into fatty acids that are incorporated into storage triglyceride (TG) (Ameer, Scandiuzzi, Hasnaina, Kalbacher, & Zaidia, 2014). In normal conditions, DNL mainly occurs in liver and adipose tissue to maintain serum TG homeostasis (Ameer et al., 2014; Bjorntorp & Sjostrom, 1987). DNL has been shown to increase abnormally in the pathogenesis of NAFLD and insulin resistance (Ameer et al., 2014; Donnelly et al., 2005). The primary regulators of DNL, including insulin and lipogenic transcription factors, namely liver X receptors, sterol regulatory element‐binding protein‐1c (SREBP‐1c), and carbohydrate response element‐binding protein, exert significant control over the de novo synthesis of fatty acids (Strable & Ntambi, 2010). Additionally, DNL is reported to be regulated by fatty acid synthase (FAS) (Ameer et al., 2014).

*Ruellia tuberosa* L. (RTL) has been demonstrated to exhibit antidiabetic, antioxidant, anti‐inflammation, and anticancer activity (Chen, Wu, Shieh, Kou, & Hsie, h C.Y., 2006; Chothani, Patel, Mishra, & Vaghasiya, 2010). Moreover, RTL may also possess the ability to regulate blood glucose and lipid balance in alloxan‐induced diabetic rats (Manikandan, Arokia, & Doss, 2010; Rajan, Kumar, Kumar, Swathi, & Haritha, 2012). Our previous study revealed that RTL ameliorates hyperlipidemia, hyperglycemia, and hyperlipidemia in high‐fat diet (HFD) and streptozotocin (STZ)‐induced T2DM rats (Ko et al., 2018, 2019). However, there are limited studies in the literature focusing on the effect of RTL on hepatic lipid metabolism in T2DM. The aim of this study is to investigate the ameliorative effect of RTL on NAFLD and lipid accumulation in liver of HFD/ STZ‐induced T2DM rat model.

## 2 MATERIALS AND METHODS

### 2.1 Preparation of RTL extracts

The stems and leaves of RTL were purchased from the Herb Light farm, Yi‐Lan County, Taiwan, in May of 2014 and identified by Prof. Wei‐Jan Huang in the College of Pharmacy, Taipei Medical University. A voucher specimen (TMU27423) was deposited in the herbarium of College of Pharmacy, Taipei Medical University. All samples were washed, dried, weighed, sliced, and freeze dried. Each 1 g dried stem or leaf was extracted with 6 ml of distilled water (RTLW) or 95% ethanol (RTLE) (1:6, w/v) individually at 4°C for 72 hr and then filtered through cheese cloth. The filtrate was further filtered twice through Whatman No. 1 filter paper before centrifuged at 4,700 × g for 20 min. The supernatant was vacuum concentrated using a rotary evaporator below 40°C. The concentrate was freeze dried into a powder and stored at −80°C until used.

### 2.2 Animals and Diets

Male Wistar rats (age 4 weeks) were purchased from the National Laboratory Animal Center, Taipei, Taiwan. The room conditions and treatment procedures were in accordance with the National Institutes of Health Guide for the Care and Use of Laboratory Animals, and all of the protocols were approved by the Institutional Animal Care and Use Committee of National Taiwan Normal University, Taipei, Taiwan (approval number 103042). The rats were maintained under standard laboratory conditions at a temperature of 23 ± 1°C and a 12‐hr light/12‐hr dark cycle, with free access to food and water for the duration of the study.

HFD/STZ‐induced T2DM rats were carried out by the method of Ma et al. (2014) with slight modifications. After 1‐week adaptation, they were fed an HFD (60% calories from fat) for 4 weeks. STZ (28 and 15 mg/kg body weight, respectively, dissolved in 0.1 M sodium citrate buffer at pH 4.5) was intraperitoneally injected into rats at the 5th and 6th weeks to induce diabetes. The diabetic rats were then fed HFD for another 6 weeks prior to experimental procedures to guarantee the stable phenomena of hyperglycemia. For the animal experimental design, the rats were divided into seven groups (each contains six rats): Group 1 consists of rats fed a normal diet for 11 weeks; Group 2 diabetic rats fed an HFD (60% calories from fat) for 11 weeks as the negative control; Group 3 diabetic rats fed an HFD and orally administered pioglitazone (Pio; 30 mg/kg body weight) daily during the last 4 weeks of the 11 weeks’ experiment as the positive control; Groups 4 and 5 diabetic rats fed an HFD and orally administered RTLW (100 or 400 mg/kg body weight, respectively) daily during the last 4 weeks of the 11 weeks’ experiment; and Groups 6 and 7 diabetic rats fed an HFD and orally administered RTLE (100 or 400 mg/kg body weight, respectively) daily during the last 4 weeks of the 11 weeks’ experiment. The body weight was monitored each week. The liver weight and adipose (epididymal and perirenal adipose tissues) weight were monitored after scarification at the end of the experiment. The livers were stored at −80°C for triglyceride determination and Western blot analysis.

### 2.3 Hepatic triglyceride assay

Hepatic triglyceride assay was carried out by Triglyceride Colorimetric Assay Kit (Cayman Chemical, Co) and performed according to the protocol.

### 2.4 Histopathological analysis

Liver tissue was removed and immediately fixed in 10% neutral phosphate‐buffered formalin solution and embedded in paraffin. Sections 4, 5 um thick were cut by a rotary microtome (Leica Microsystems, Wetzlar, Germany) and stained by hematoxylin–eosin (H & E). The stained specimens were observed and photographed by utilizing an upright digital imaging microscope (Zeiss Axioplan 2).

### 2.5 Liver and epididymal adipose tissue protein preparation

The liver (0.5 g) or epididymal adipose (0.05g) was homogenized with lysis buffer (0.2% Triton X‐100, 5 mmol/l EDTA, and 1 mmol/l phenylmethylsulfonyl fluoride) at 4°C for 2 min and then centrifuged (10,000 × g, 20 min, 4°C) to acquire the supernatant. The protein concentration in the cell extract was determined using a Bio‐Rad protein assay.

### 2.6 Western blot analysis

The Western blot was adopted by Huang, Wen‐Chang Chang, Wu, Shih, and Shen (2016) with slight modification. Briefly, aliquots of the extract were evaluated for the expression of sterol regulatory element‐binding protein‐1 (SREBP1) and FAS in liver. The samples were subjected to gel electrophoresis and further electrotransferred to a polyvinylidene difluoride membrane. The membrane was incubated with blocking buffer and probed with anti‐SREBP1 and anti‐FAS (1:1000) overnight at 4°C. The intensity of the blots probed with a 1:5000 dilution of mouse monoclonal antibody against α‐tubulin was used as a control. The membrane was washed in phosphate‐buffered saline with Tween 20 (PBST), shaken in a solution of horseradish peroxidase‐conjugated anti‐mouse IgG or anti‐rabbit IgG secondary, and incubated in enhanced chemiluminescence reagent. Autoradiography was scanned and analyzed using a UVP Biospectrum image system (Level, Cambridge, UK).

### 2.7 Statistical analysis

Results are presented as the mean ± standard deviation (*SD*), which was analyzed statistically with SAS Version 9.4 (SAS Institute Inc, Cary, NC, USA) using one‐way ANOVA and Duncan's new multiple range tests. All comparisons were made relative to the normal group, where *p* < .05 is considered to be statistically significant.

## 3 RESULTS

### 3.1 Effect of RTL extracts on organ weight in HFD/STZ‐induced T2DM rats

Table 1 shows the changes of organ weight in rats after 4 weeks of administration of RTL extracts. Liver, perirenal, and epididymal adipose tissues from rats were acquired and weighed after sacrifice. HFD fed plus STZ injection rats (DM group) caused 23% and triple increase of relative liver and adipose tissue weights, respectively, in comparison to normal rats (*p* < .05; Table 1).

**TABLE 1:** The selected organ weight of high‐fat diet and streptozotocin‐induced type 2 diabetic rats fed with *Ruellia tuberosa* L. (RTL) extracts for 4 weeks

	*N*	DM	DM + Pio	DM + W100	DM + W400	DM + E100	DM + E400
Liver weight (g/rat)	13.07 ± 2.46^b^	16.66 ± 0.83^a^	14.67 ± 1.68^ab^	15.30 ± 0.84^a^	14.74 ± 1.71^ab^	15.41 ± 1.51^a^	16.19 ± 1.13^a^
Relative liver weight (g/100g body weight)	2.63 ± 0.20^cd^	3.23 ± 0.22^a^	2.53 ± 0.11^d^	2.99 ± 0.09^b^	2.83 ± 0.24^bc^	2.84 ± 0.24^bc^	2.86 ± 0.24^bc^
Epididymal and perirenal fat weight (g/rat)	11.85 ± 3.29^c^	40.56 ± 4.21^a^	33.00 ± 6.43^b^	31.98 ± 6.48^b^	28.50 ± 6.64^b^	32.32 ± 10.36^c^	34.38 ± 8.32^ab^
Relative fat weight (g/100g body weight)	2.44 ± 0.55^c^	7.49 ± 0.57^a^	5.40 ± 0.74^b^	6.19 ± 0.77^b^	5.72 ± 1.00^b^	6.03 ± 1.55^b^	6.16 ± 1.17^b^

Note

Normal: Normal diet; DM: high‐fat diet (HFD; 60% fat) plus STZ (28 mg/kg body weight, i.p.) induced diabetic rats; DM + Pio: DM rats gavaged with pioglitazone (30 mg/kg body weight) for 4 weeks; DM + W100: DM rats gavaged with RTL water extract (100 mg/kg body weight) for 4 weeks; DM + W400: DM rats gavaged with RTL water extract (400 mg/kg body weight) for 4 weeks; DM + E100: DM rats gavaged with RTL ethanol extract (100 mg/kg body weight) for 4 weeks; DM + E400: DM rats gavaged with RTL ethanol extract (400 mg/kg body weight) for 4 weeks. Values were calculated as the mean ± *SD*, *n* = 6 for each group. Notes: a–c letters = significant differences among all samples tested in the same row (*p* < .05).

### 3.2 Effect of RTL extracts on fat accumulation in liver of HFD/STZ‐induced T2DM rats

**FIGURE 1:**
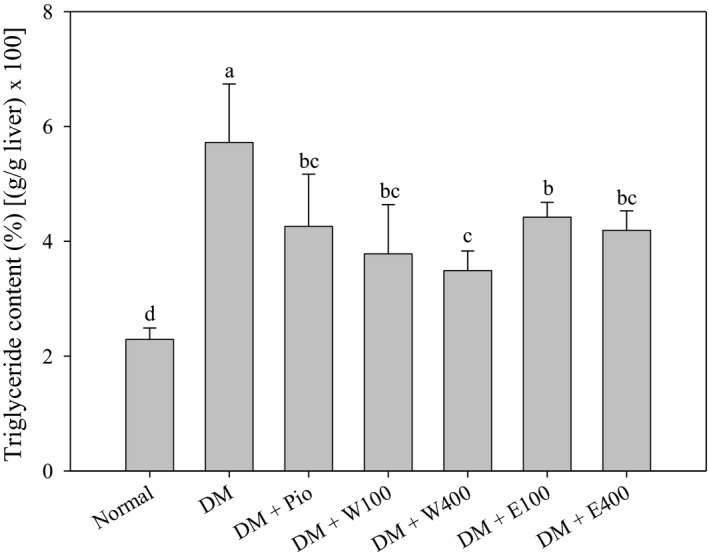
The hepatic triglyceride content of high‐fat diet and streptozotocin‐induced type 2 diabetic rats fed with *Ruellia tuberosa* L. (RTL) extracts for 4 weeks. Normal: Normal diet; DM: high‐fat diet (HFD; 60% fat) plus STZ (28 mg/kg body weight, i.p.) induced diabetic rats; DM + Pio: DM rats gavaged with pioglitazone (30 mg/kg body weight) for 4 weeks; DM + W100: DM rats gavaged with RTL water extract (100 mg/kg body weight) for 4 weeks; DM + W400: DM rats gavaged with RTL water extract (400 mg/kg body weight) for 4 weeks; DM + E100: DM rats gavaged with RTL ethanol extract (100 mg/kg body weight) for 4 weeks; DM + E400: DM rats gavaged with RTL ethanol extract (400 mg/kg body weight) for 4 weeks. Values were calculated as the mean ± *SD*, *n* = 6 for each group. Notes: a–d letters = significant differences among all samples tested (*p* < .05)

**FIGURE 2:**
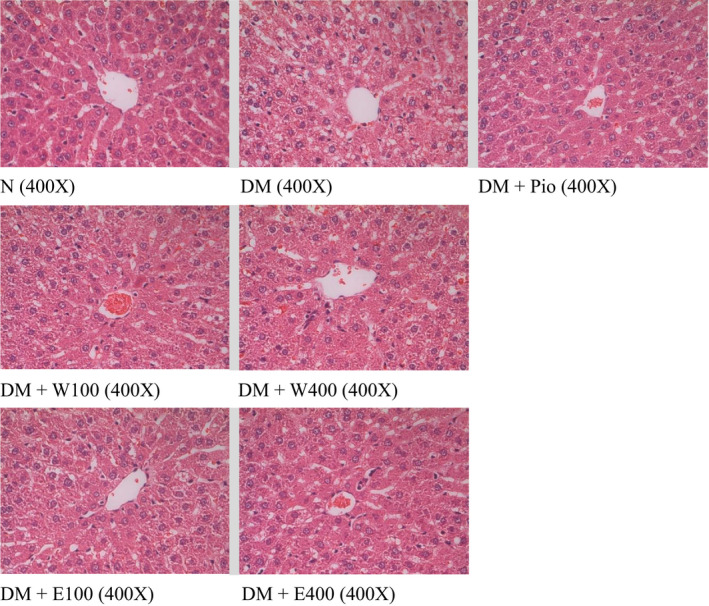
The liver histopathological alteration of high‐fat diet and streptozotocin‐induced type 2 diabetic rats fed with *Ruellia tuberosa* L. (RTL) extracts for 4 weeks. (H&E stain, 400X). Normal: Normal diet; DM: high‐fat diet (HFD; 60% fat) plus STZ (28 mg/kg body weight, i.p.) induced diabetic rats; DM + Pio: DM rats gavaged with pioglitazone (30 mg/kg body weight) for 4 weeks; DM + W100: DM rats gavaged with RTL water extract (100 mg/kg body weight) for 4 weeks; DM + W400: DM rats gavaged with RTL water extract (400 mg/kg body weight) for 4 weeks; DM + E100: DM rats gavaged with RTL ethanol extract (100 mg/kg body weight) for 4 weeks; DM + E400: DM rats gavaged with RTL ethanol extract (400 mg/kg body weight) for 4 weeks

Figure 1 showed that treatment of HFD and STZ caused 2.5 times increase in hepatic TG levels in type 2 diabetic rats (5.72 ± 1.04%), when compared with normal group (2.29 ± 0.20%) (*p* < .05). Administration of RTLW and RTLE may ameliorate NAFLD and hepatic TG accumulation in type 2 diabetic rats. RTLW at dosage of 400mg/ kg body weight led to the maximum decrease by 38.9% when compared with that of DM group (*p* < .05). The results of histochemical stain revealed that HFD and STZ treatment caused hypertrophy and tiny vacuoles on the inside of liver cell, also known as slight steatosis (Figure 2). RTL may significantly improve the progression of hypertrophy and tiny vacuoles.

### 3.3 Effect of RTL extracts on the DNL‐associated protein expression of fatty acid metabolism in liver of HFD/STZ‐induced type 2 T2DM rats

**FIGURE 3:**
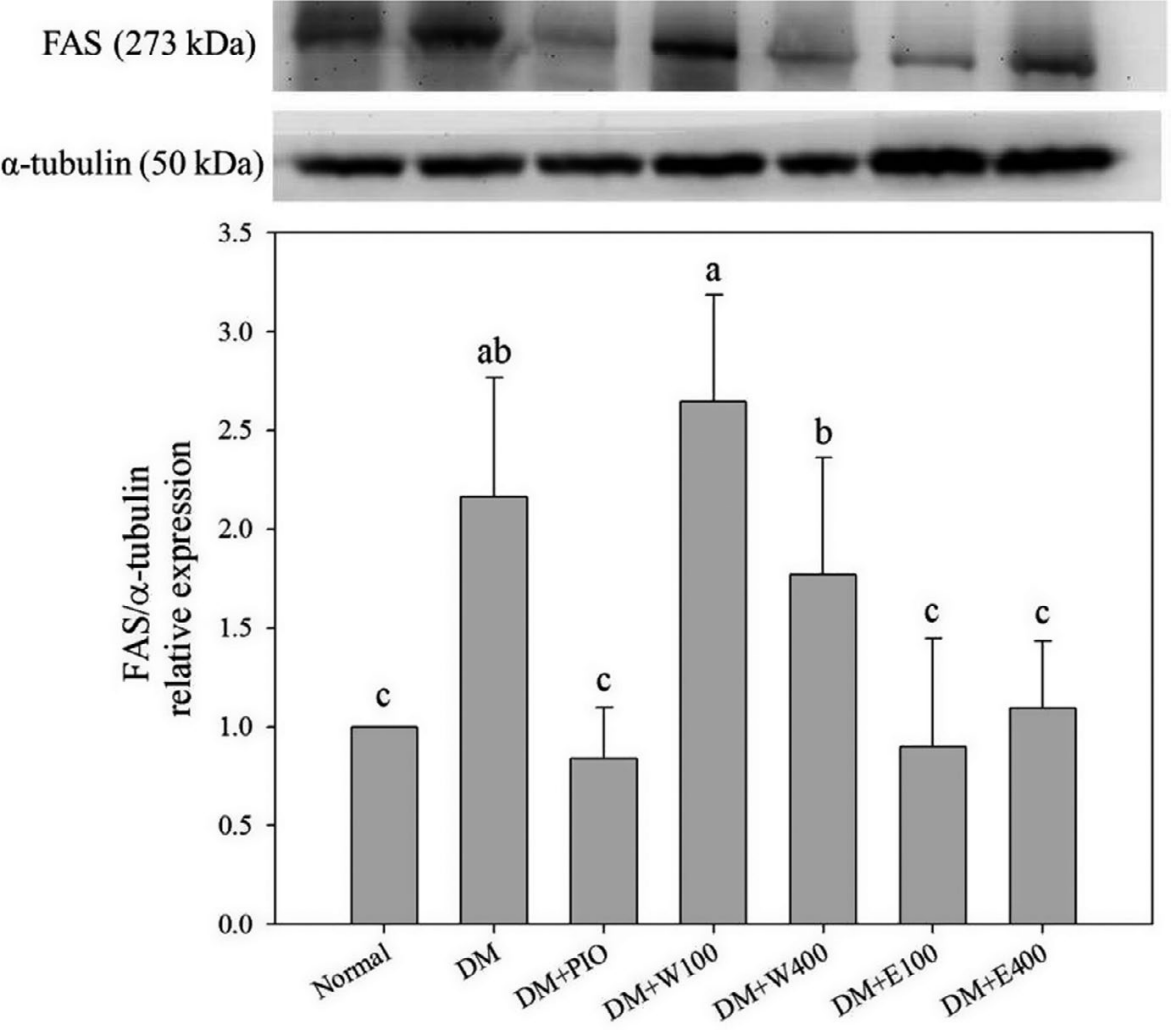
Protein expression of hepatic FAS in high‐fat diet and streptozotocin‐induced type 2 diabetic rats fed with *Ruellia tuberosa* L. (RTL) extracts for 4 weeks. Normal: Normal diet; DM: high‐fat diet (HFD; 60% fat) plus STZ (28 mg/kg body weight, i.p.) induced diabetic rats; DM + Pio: DM rats gavaged with pioglitazone (30 mg/kg body weight) for 4 weeks; DM + W100: DM rats gavaged with RTL water extract (100 mg/kg body weight) for 4 weeks; DM + W400: DM rats gavaged with RTL water extract (400 mg/kg body weight) for 4 weeks; DM + E100: DM rats gavaged with RTL ethanol extract (100 mg/kg body weight) for 4 weeks; DM + E400: DM rats gavaged with RTL ethanol extract (400 mg/kg body weight) for 4 weeks. Values were calculated as the mean ± *SD*, *n* = 6 for each group. Notes: a–c letters = significant differences among all samples tested (*p* < .05)

**FIGURE 4:**
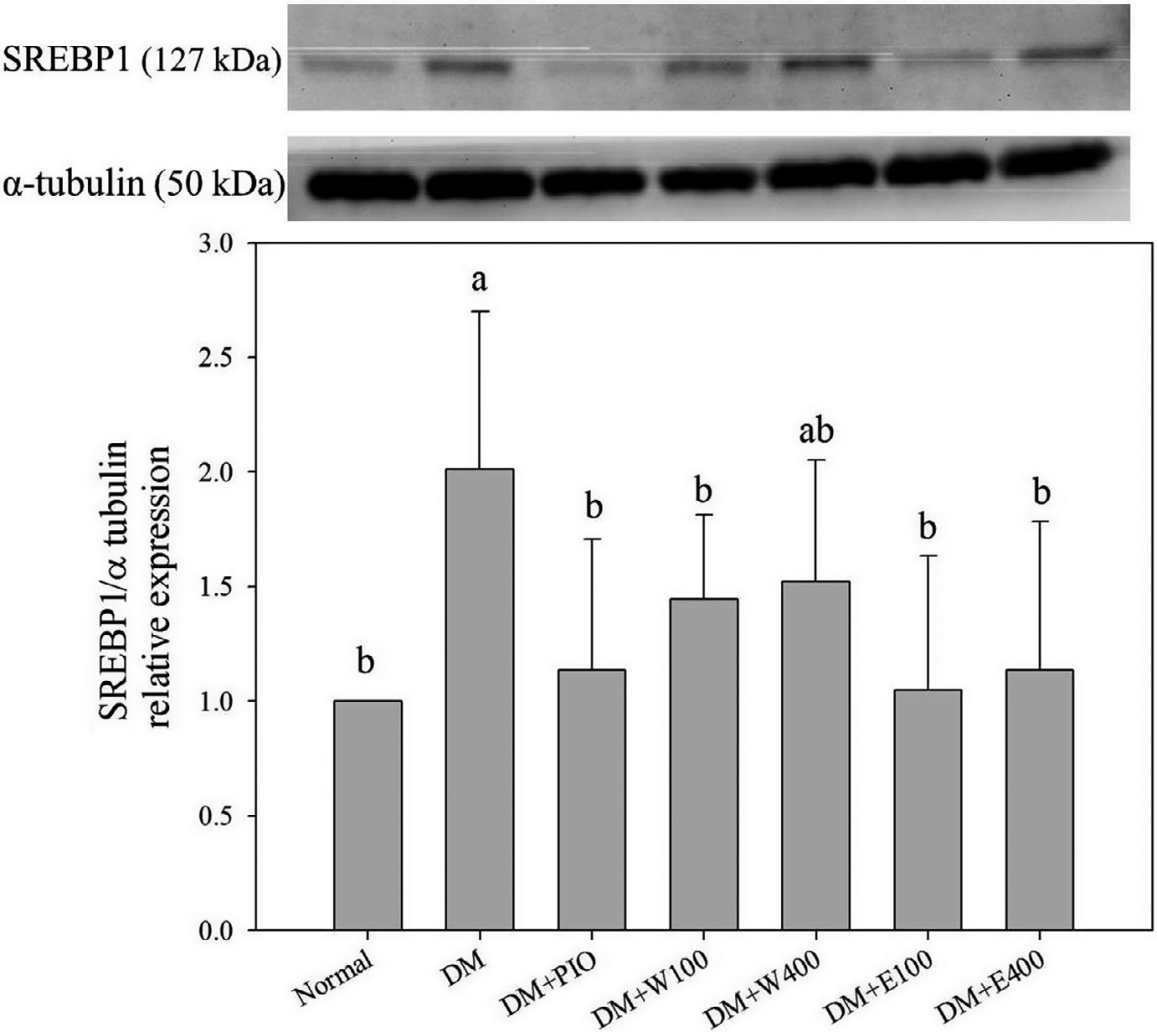
Protein expression of hepatic SREBP1 in high‐fat diet and streptozotocin‐induced type 2 diabetic rats fed with *Ruellia tuberosa* L. (RTL) extracts for 4 weeks. Normal: Normal diet; DM: high‐fat diet (HFD; 60% fat) plus STZ (28 mg/kg body weight, i.p.) induced diabetic rats; DM + Pio: DM rats gavaged with pioglitazone (30 mg/kg body weight) for 4 weeks; DM + W100: DM rats gavaged with RTL water extract (100 mg/kg body weight) for 4 weeks; DM + W400: DM rats gavaged with RTL water extract (400 mg/kg body weight) for 4 weeks; DM + E100: DM rats gavaged with RTL ethanol extract (100 mg/kg body weight) for 4 weeks; DM + E400: DM rats gavaged with RTL ethanol extract (400 mg/kg body weight) for 4 weeks. Values were calculated as the mean ± *SD*, *n* = 6 for each group. Notes: a–b letters = significant differences among all samples tested (*p* < .05)

In the present study, treatment of HFD and STZ caused 1.2 times increase in the expression of FAS as compared to that of N group (*p* < .05; Figure 3). The expression of FAS was decreased by 61.4%, 58.5%, and 49.5% in DM + PIO, DM + E100, and DM + E400 group, respectively, as compared with DM group after treatment of PIO or RTLE (Figure 3). The expression of hepatic SREBP1 was increased by 101.2% in DM group when compared with N group (*p* < .05; Figure 4). Enhanced expressions of hepatic SREBP1 were declined by 43.6%, 28.2%, 24.5%, 47.9%, and 43.6% in DM + PIO, DM + W100, DM + W400, DM + E100, and DM + E400 group, respectively, when compared with DM group (*p* < .05; Figure 4).

## 4 DISCUSSION

Jensen, Caruso, Heiling, and Miles (1989) reported that HFD enhances TG and free fatty acids (FFA) level in serum, which could lead to adipocyte hypertrophy, promote secretion of tumor necrosis factor‐α and insulin‐like growth factor, and consequently induce the growth of adipocytes. Pioglitazone was proved to promote adipocyte differentiation, reduce urine sugar, increase body fluid retention and appetite resulting in weight gain (Ghosh & Dey, 2011). Excessive fat intake may cause imbalance of lipid metabolism in liver, which also promote liver fat accumulation and tissue hypertrophy (Puigserver & Rodgers, 2006). Patients with NAFLD usually accompany the progression of hepatomegaly (Zeng et al., 2008). Results of this study revealed that RTLW and RTLE significantly reduced relative liver and adipose tissue weights in HFD/STZ‐induced diabetic rats.

Dietary fat is decomposed by lipoprotein lipase in intestine and transported to adipose tissue for storage. TG in adipose tissue is degraded into glycerol and FFA by hormone‐sensitivity lipase, resulting in elevated FFA level. FFA was integrated with serum albumin and then transported to liver to stimulate the secretion of VLDL (Zechner, Strauss, Haemmerle, Lass, & Zimmermann, 2005). Insulin resistance has been proved to cause abnormal regulation of lipolysis in peripheral adipocytes, leading to massive release of FFA into blood (Petersen & Shulman, 2006). Our previous study has revealed that RTLW and RTLE may alleviate hyperglycemia and hyperlipidemia via improving insulin resistance in HFD/STZ‐induced type 2 diabetic rats (Chang et al., 2018; Ko et al., 2019). In the normal condition, hepatic triglyceride of less than 5% is derived from endogenous lipid synthesis in the liver. Excessive fat intake from high‐fat diet may deposit in adipose tissue and organs as the form of TG, resulting in NAFLD (Ji, Zhao, Leng, Liu, & Jiang, 2011) when fat accumulation is high than 5% in liver tissue (Loomba & Sanya, 2013). Fat accumulation in liver was also considered an indicator of hepatic insulin resistance (Kotronen & Yki‐Järvinen, 2008). Obese insulin resistance may increase the risk for abnormal accumulation of fat through induction of lipid metabolism disorder in liver (Loomba & Sanya, 2013). The result of this study elucidated that RTL extracts may decrease hepatic fat accumulation and alleviate the pathogenesis of NAFLD via ameliorating lipid metabolism disorder in liver.

Hepatic DNL is recognized as the biochemical process of synthesizing fatty acid from acetyl‐CoA subunits due to excess carbohydrate (Abraham, Rabi, Francis, Priya, & M., Natarajan, K., Amaladass, A., 2016). The metabolism pathway of FFA in liver is to incorporate TG into storage via esterification. TG could be responsible for energy production via β‐oxidation (Ameer et al., 2014). High‐carbohydrate diet was reported to activate a lipogenic response in the liver tissue (Ameer et al., 2014; Strable & Ntambi, 2010). In addition to high‐carbohydrate diets, HFD has been shown to induce the hepatic expression of lipogenic enzymes involved in the de novo synthesis of fatty acids and lipids (Strable & Ntambi, 2010). DNL or de novo synthesis of fatty acids was reported to be regulated by namely FAS. FAS plays a key role in the conversion of malonyl‐CoA into palmitate for fatty acid synthesis. A previous study revealed that increase of FAS expression resulted in increase of DNL in livers from diabetic rats (Abraham et al., 2016). SREBP1 was reported to positive regulated FAS expression, which caused TG synthesis (Goedekeet al., 2018). HFD has been proved to cause TG accumulation via increasing SREBP‐1c and FAS expression (Ji et al., 2011). This study could be comprehended that RTLW decreased FAS expressions via suppressing the expression of SREBP1 to reduce TG production in liver and subsequently ameliorate NAFLD in HFD/STZ‐induced T2DM rats.

## 5 CONCLUSIONS

The present study first demonstrated that RTLW may decrease DNL via downregulating SREBP1 and FAS expressions in liver, while inhibiting the accumulation of hepatic TG and formation of NAFLD in HFD/STZ‐induced diabetic rats. We suggested that RTL may be a potential therapy for amelioration on hepatic dyslipidemia and steatosis in T2DM rats. The purification and identification of active components in RTL extracts are currently on the way for further investigation in our laboratory.

## CONFLICT OF INTEREST

The authors declare that they do not have any conflict of interest.

## AUTHORS CONTRIBUTIONS

SCS participated in the design of the study and wrote the protocol and the manuscript. DWH and YML participated in the design and discussion of the experiments and the writing of the manuscript. RTL analyzed the results and jointly wrote the manuscript. DWH, YML, and JSBW conducted literature searches. WCC, CYL, and YFC carried out animal experiments.

## ETHICS STATEMENT

The study was conducted in accordance with the ethical guidelines of the Institutional Animal Care and Use Committee of National Taiwan Normal University, Taipei, Taiwan (approval no. 103042).
